# Dynamic reorganization of flotillins in chemokine-stimulated human T-lymphocytes

**DOI:** 10.1186/1471-2121-12-28

**Published:** 2011-06-22

**Authors:** Sarah Affentranger, Sibylla Martinelli, Jonas Hahn, Jérémie Rossy, Verena Niggli

**Affiliations:** 1 Dept. of Pathology, University of Bern, CH-3010 Bern, Switzerland; 2 Present address: Membrane Biology Group, Centre for Vascular Research, University of New South Wales, Lowy Cancer Research Centre, Sydney NSW 2052, Australia

## Abstract

**Background:**

Different types of membrane microdomains (rafts) have been postulated to be present in the rear and front of polarized migrating T-lymphocytes. Disruption of rafts by cholesterol sequestration prevents T-cell polarization and migration. Reggie/flotillin-1 and -2 are two highly homologous proteins that are thought to shape membrane microdomains. We have previously demonstrated the enrichment of flotillins in the uropod of human neutrophils. We have now investigated mechanisms involved in chemokine-induced flotillin reorganization in human T-lymphocytes, and possible roles of flotillins in lymphocyte polarization.

**Results:**

We studied flotillin reorganization and lateral mobility at the plasma membrane using immunofluorescence staining and FRAP (fluorescence recovery after photobleaching). We show that flotillins redistribute early upon chemokine stimulation, and form very stable caps in the uropods of human peripheral blood T-lymphocytes, colocalizing with the adhesion molecule PSGL-1 and activated ezrin/radixin/moesin (ERM) proteins. Chemokine-induced formation of stable flotillin caps requires integrity and dynamics of the actin cytoskeleton, but is not abolished by inhibitors suppressing Rho-kinase or myosin II activity. Tagged flotillin-2 and flotillin-1 coexpressed in T-lymphocytes, but not singly expressed proteins, colocalize in stable caps at the tips of uropods. Lateral mobility of coexpressed flotillins at the plasma membrane is already partially restricted in the absence of chemokine. Incubation with chemokine results in almost complete immobilization of flotillins. Capping is abolished when wild-type flotillin-1 is coexpressed with a mutant of flotillin-2 (G2A) that is unable to interact with the plasma membrane, or with a deletion mutant of flotillin-2 that lacks a putative actin-binding domain. Wild-type flotillin-2 in contrast forms caps when coexpressed with a mutant of flotillin-1 unable to interact with membranes. Transfection of T-lymphocytes with flotillin-2-G2A reduces cell polarization and uropod recruitment of endogenous flotillin-1 and PSGL-1.

**Conclusions:**

Our data suggest that stable flotillin cap formation in the rear of polarized T-lymphocytes requires flotillin heterooligomer formation, as well as direct F-actin interactions of flotillin-2 and raft/membrane association of flotillin-2, but not -1. Our data also implicate flotillin-rich actin-dependent membrane microdomains in T-lymphocyte uropod formation.

## Background

Adaptive immune cells such as T-cells, continuously travel through the tissues, using amoeboid locomotion. This enables these cells to rapidly recognize foreign antigens, to stimulate antibody production and to destroy virally infected cells or tumor cells. T-cell polarization and directional migration is a complex, not yet well understood process. Certainly it involves a functional cytoskeleton, reversible actin polymerization in the front and myosin-dependent contractility in the rear of a migrating, polarized cell [1-3].

Polarization of leukocytes requires segregation and activation of specific signaling and cytoskeletal molecules in the retracting rear (uropod) and motile forward moving part (front) of the cells [4]. Localized positive feedback loops and inhibitory effects of front signaling pathways on rear signaling and vice versa are thought to reinforce this biochemical and structural cell polarization [5]. The compartmentalization of signaling molecules could be stabilized by formation of plasma membrane microdomains ("rafts") that are thought to organize signaling systems in the membrane [6].

Interestingly current evidence suggests the presence of different types of membrane microdomains in the front and tail of polarized migrating leukocytes. The raft-resident lipid GM1 ganglioside is clustered in the tail of polarized migrating T-lymphocytes and neutrophils, whereas another raft marker, the ganglioside GM3, is present in the front of these cells [6]. Experiments using depletion of cellular cholesterol by treating cells with methyl-β-cyclodextrin (MβCD) indicate indeed a crucial role for cholesterol-dependent rafts in neutrophil and T-lymphocyte polarization and migration [7-9]. However little is known on raft organization in migrating leukocytes.

Reggie/flotillin-1 and -2 are two highly homologous proteins whose enrichment in membrane microdomains has been ubiquitously observed. Flotillins, peripheral membrane-associated proteins, are thought to be involved in structuring membrane microdomains and have been implicated in the delivery of membranes and membrane proteins to cell contact sites, regenerating axons, growth cones etc [10,11]. Flotillins are thus attractive candidates for the structuring of membrane microdomains in T-cells that lack caveolins. Membrane-associated caps of flotillins have been observed in T-cell lines such as Jurkat cells, human T lymphoblasts and monocytes [12-14]. However the functional role of flotillins in T-cell migration has not yet been explored except for the report by Giri et al. [15] which suggests a role of flotillin-1 in chemotaxis, adhesion, calcium mobilization and Rac activation in freshly isolated human T-cells. The role of flotillin-2 was not addressed in this study.

We have recently shown that flotillin-1 and -2 interact and, upon stimulation with chemotactic peptide, rapidly form membrane caps and at later time points accumulate in the uropods of polarized primary human neutrophils, preceding capping of other uropod proteins such as ezrin/radixin/moesin (ERM). Capping of endogenous flotillins in human neutrophils depends on actin dynamics but does not require myosin II activity. Flotillin capping and redistribution to the uropod matches temporally and spatially that of P-selectin glycoprotein ligand 1 (PSGL-1), and flotillins interact with PSGL-1 in primary human neutrophils. PSGL-1 is not required for the capping of flotillins [16]. This work suggests an important role of flotillins in structuring the neutrophil uropod. Recent work by Ludwig et al. using neutrophils from flotillin 1 knockout mice confirmed and extended our work, showing that the lack of flotillin-1, which leads to a reduction of flotillin-2 and its displacement from rafts, results in impairment of murine neutrophil uropod formation and migration through matrigel [17]. We have now studied mechanisms of reorganization of flotillins in polarizing T-lymphocytes. We provide novel data on the regulation of stimulus-dependent uropod recruitment of flotillins by the cytoskeleton and by raft-association, on the stimulus-dependent immobilization of flotillins and on the functional roles of these proteins in polarization of T-lymphocytes.

## Methods

### Materials

Materials and suppliers: stromal cell-derived factor 1 (SDF-1 = CXCL12): Peprotech, Paris, France. Y-27632, Jasplakinolide: Calbiochem, Darmstadt, Germany. Latrunculin A: Alexis Biochemicals, Switzerland. Blebbistatin: Tocris Bioscience, UK. Bovine serum albumin (BSA): Serva, Germany. Lysolecithin (L-α-lysophosphatidylcholine): Sigma. Ficoll-Paque™ Plus: GE Healthcare Bio-Sciences AB, Uppsala, Sweden. Gey's solution contained 138 mM NaCl, 6 mM KCl, 1 mM MgSO_4_, 1.1 mM CaCl_2_, 100 μM EGTA, 1 mM Na_2_HPO_4_, 5 mM NaHCO_3_, 5.5 mM glucose and 20 mM HEPES (pH 7.4).

### Antibodies

A polyclonal anti-CD3 antibody (Cat. No. RM-9107) was obtained from NeoMarkers, Fremont, CA, USA. Polyclonal anti-phospho Ezrin (Thr567)/ Radixin (Thr564)/ Moesin (Thr558) antibody (Cat. No. 3141S) was from Cell Signaling, MA, USA. Polyclonal anti-flotillin-2 (Cat. No. sc-25507) was from Santa Cruz Biotechnology, USA. A monoclonal anti-actin antibody (Cat. No. 010056) was from Bio-Science Products AG, Switzerland. Monoclonal murine antibodies directed against flotillin-1 (Cat. No. F65020), flotillin-2 (Cat. No. E35820) and PSGL-1 (Cat. No. 556053) were obtained from Transduction Laboratories/BD Pharmingen, Germany. A monoclonal murine antibody directed against the protein tag YPYDVPDYA (HA) (Cat. No. H9658) was obtained from Sigma, and a polyclonal rabbit anti-HA antibody (Cat. No. 561) was obtained from MBL, Japan. The Alexa 488-conjugated goat-anti-rabbit (Cat. No. A11008), Alexa-568-conjugated goat anti-rat (Cat. No. A11077) and goat anti-mouse IgG antibodies (Cat. No. A11001) were from Molecular Probes, OR, USA.

### Isolation and culture of human T-lymphocytes

Peripheral blood lymphocytes were isolated from buffy coats of healthy donor blood (Central Laboratory of the Swiss Red Cross, Bern, Switzerland). Buffy coats were diluted 1:4 with phosphate-buffered saline (PBS) lacking calcium and magnesium. 30 ml of this suspension was layered over 15 ml of Ficoll-Paque™ Plus and centrifuged at 500 × g for 25 minutes at room temperature. The mononuclear cell layer was removed, washed in Hank's buffered salt solution and resuspended in RPMI containing 10% fetal calf serum (FCS) (4 × 10^6 ^cells/ ml). Cells were activated for 2 days by culturing with 1 μg/ml phytohemagglutinin (PHA), followed by culturing for 5 more days in fresh RPMI containing 50 U/ml IL-2. These cells are referred to as "T-lymphoblasts". T-lymphoblasts were used between day 7 and 20 after isolation from the buffy coats. The preparations contained > 86% CD3-positive cells. Prior to some experiments, when indicated in the figure legends, T-lymphoblasts were incubated in culture medium lacking IL-2 at 37°C for 14-18 hours. Medium was changed every 2-3 days.

For some experiments, freshly isolated T-lymphocytes were used as indicated. These cells were isolated from buffy coats using the Pan T Cell Isolation Kit II (Miltenyi Biotec) and separation on LD columns (Miltenyi Biotec) according to the manufacturers instructions. Briefly, mononuclear cells obtained from buffy coats as described above were incubated with a cocktail of biotin-conjugated antibodies against CD14, CD16, CD19, CD36, CD56, CD123, and CD235a (glycophorin A). These cells were subsequently depleted from the cell suspension using anti-biotin MicroBeads. The resulting cell suspension contained >95% T-lymphocytes as assessed using anti-CD3 staining. The cells were used after over night incubation in RPMI with 10% FCS at 37°C and 5% CO_2_.

### Immunofluorescence staining

Cells were incubated in suspension in plastic tubes in Gey's buffer lacking calcium and magnesium at 37°C with agitation (750 rpm) without or with inhibitors and stimuli (4-8 × 10^6 ^cells/ml, 500 μl per assay) as indicated in the Figure legends. After the incubation cells were fixed by addition of 0.5 ml 20% trichloroacetic acid (TCA) prewarmed at 37°C and a 5 minute incubation, or (for visualization of EGFP-tagged proteins) by incubation with 3.7% (final) paraformaldehyde (PFA) for 10 minutes at 37°C. In part of the experiments, cells resuspended in Gey's buffer containing calcium and magnesium were plated on glass coverslips coated with 20 μg/ml fibronectin, followed by incubation without or with inhibitors and stimuli, and TCA fixation. Cells then were washed with PBS, cytocentrifuged, permeabilized with lysolecithin (60 μg/ml) for 10 minutes at RT and washed with PBS. Cells were then blocked for 30 minutes with blocking buffer (PBS containing 5% BSA and 10% normal goat serum). Subsequently cells were incubated with the primary antibody diluted in PBS, 0.5% BSA at room temperature for one hour (dilutions: rabbit anti-flotillin-2, murine anti-flotillin-1, rabbit anti-CD3, all diluted 1:100; murine anti-flotillin-2, rabbit anti-P-ERM, murine anti- human PSGL-1, murine anti-actin: all diluted 1:400; rabbit or murine anti-HA-antibody: diluted 1:500). The preparations were then rinsed once with TBST (50 mM Tris, pH 7.4, 150 mM NaCl, 0.05% Tween) and twice with PBS and blocked again for 15 minutes with blocking buffer. Cells were then incubated with the appropriate secondary antibodies (Alexa 488-conjugated goat-anti-rabbit or goat anti-mouse IgG antibodies diluted 1:500) at room temperature for 1 hour, followed by washing once with TBST and twice with PBS. Images were taken with a confocal laser scanning microscope Olympus Fluoview FV1000-IX81, 63× oil immersion objective. Flotillin-enriched aggregates located at the plasma membrane of at least 1 μm or larger (maximally approximately 4 μm) were defined as "caps".

### Constructs

Constructs encoding for flotillin-2 or flotillin-1 C-terminally tagged with EGFP and flotillin-2 or flotillin-1 C-terminally tagged with mCherry were prepared as described [16].

Flotillin-1 or -2 cloned into the plasmid pEGFP-N1 [16] were used as a PCR template to generate the mutants flotillin-1-C34A and flotillin-2-G2A. The single-point mutations were inserted by PCR using specific primers, and the products were cloned into the vectors pEGFP-N1 or pmCherry-N1 (ClonTech Laboratories). The deletion mutant flotillin-2-R1MCT [18] was obtained by PCR using flotillin-2 cloned into pEGFP-N1 as template and primers introducing a HindIII restriction site instead of the SPFH domain (aa 30-184). The fragments were then subcloned into the vector pEGFP-N1.

For preparation of constructs encoding for HA-tagged proteins, the plasmids described above containing flotillin-1, flotillin-2, flotillin-1-C34A and flotillin-2-G2A were used as a PCR template. Then the PCR products were cloned into the phCMV3 Vector (Genlantis), generating constructs encoding flotillins C-terminally tagged with HA.

### Transient transfections of T-lymphocytes

For transfections, 5-8 × 10^6 ^T-lymphoblasts or freshly isolated T-lymphocytes were resuspended in 100 μl human T cell nucleofector solution (Amaxa, Köln, Germany) diluted 1:2 with PBS and 2-3 μg of plasmid DNA were added. Then, the cell suspension and the plasmid DNA were transferred to a cuvette and Nucleofection was carried out (Amaxa Nucleofector, program T-23 for T-lymphoblasts, program U-14 for freshly isolated T-lymphocytes). Immediately, 500 μl of medium with 20% FCS was added and the cells were transferred to a prewarmed 12-well plate containing 2.5 ml of medium with 20% FCS, followed by incubation at 37°C in a CO_2 _incubator for 4 hours. Transfected cells were then washed and resuspended in Gey's solution, followed by stimulation with SDF-1 (40 ng/ml) for 15 minutes at 37°C, cell fixation and immunofluorescence staining. In some experiments, T-lymphoblasts were incubated for 24 hours in the absence of IL-2 at 37°C prior to transfections. Five hours after transfection, IL-2 was added to the medium. 22 hours after transfection, cells were incubated with SDF-1 (40 ng/ml) in Gey's medium lacking calcium and magnesium for 15 min, followed by fixation and staining.

### Fluorescence recovery after photobleaching (FRAP) experiments

T-lymphocytes were transfected with either flotillin-2-EGFP alone or with flotillin-2-EGFP and flotillin-1-mCherry together, followed by an incubation for 4 hours at 37°C in a CO_2 _incubator and a 15 minutes incubation in the absence or presence of 40 ng/ml SDF-1 in Gey's medium with 5% BSA. An aliquot of 5 μl of cell suspension (10 × 10^6 ^cells/ml) was then immediately placed on a glass slide, covered with a glass coverslip and sealed with hot paraffin. Live-cell confocal imaging was performed at room temperature with a confocal laser scanning microscope Olympus Fluoview FV1000-IX81, 63× oil immersion objective. A picture of the cells was taken 2 seconds before bleaching of the EGFP or mCherry moiety. A circular region of interest (ROI) of 2.5 μm in diameter was continuously bleached at 8% of full laser power (473 nm for EGFP; 559 nm for mCherry) for 12 seconds. Seven seconds after the completion of bleaching the first frame was taken. Fluorescence recovery was observed for 140 seconds (20 frames) at 0.5-1% laser power. For analysis the fluorescence of the bleached ROIs, corrected for the reduction in fluorescence of control ROIs located outside of the bleached area in the same cell which occurs due to taking of frames, and normalized to the initial fluorescence in the ROIs before bleaching, were plotted against time. The mobile fraction was calculated after correction for bleaching of the control ROIs according to M_F _= (F_FR_-F_0_)/F_i_-F_0_) where F_FR _is the average fluorescence after full recovery, F_0 _is the fluorescence immediately after the bleach and F_i _is the fluorescence just before photobleaching.

### Statistics

Differences between data were analysed with the Student's t test, with a *P *value < 0.05 considered significant. Data correspond to the mean ± s.e.m.

## Results

### Flotillin-1 and -2 redistribute rapidly to the uropod upon chemotactic stimulation of human T-lymphoblasts

**Figure 1:**
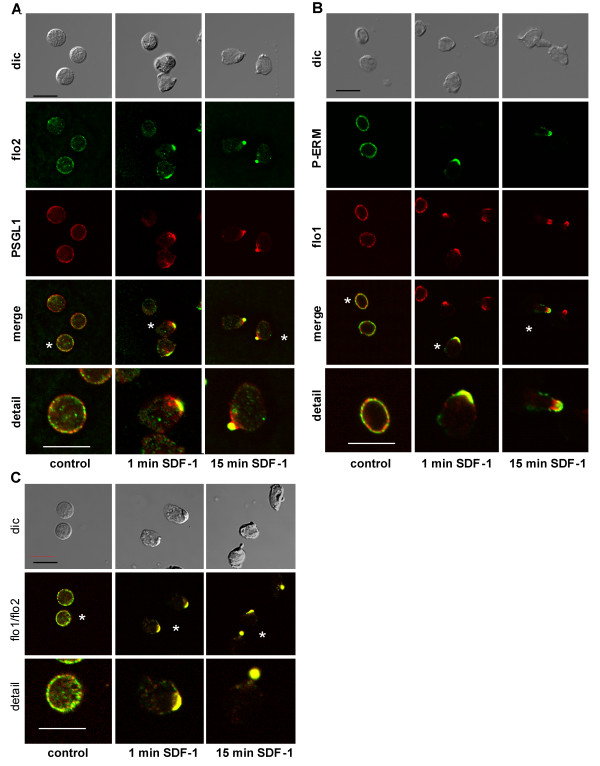
**Redistribution of flotillin-1 and -2, PSGL-1 and P-ERM upon stimulation of human T-lymphoblasts in suspension**. (A-C) After an overnight incubation in the absence of IL-2, cells were incubated either for 45 min at 37°C (control) or were stimulated with 40 ng/ml SDF-1 for 1 or 15 minutes after a 44 or 30 minutes preincubation at 37°C (total incubation time 45 minutes). Cells were then fixed with 10% TCA and double-labeled for flotillin-2 (flo2; rabbit Ab) and PSGL-1 (murine Ab) in (A) or for flotillin-1 (flo1; murine Ab) and P-ERM (rabbit Ab) in (B). or for flotillin-1 (murine Ab) and flotillin-2 (rabbit Ab) in (C). Asterisks indicate cells shown enlarged in the panels labelled "detail". Dic; differential interference contrast. Bar, 10 μm. Note that in panels C overlays only are shown.

**Figure 2:**
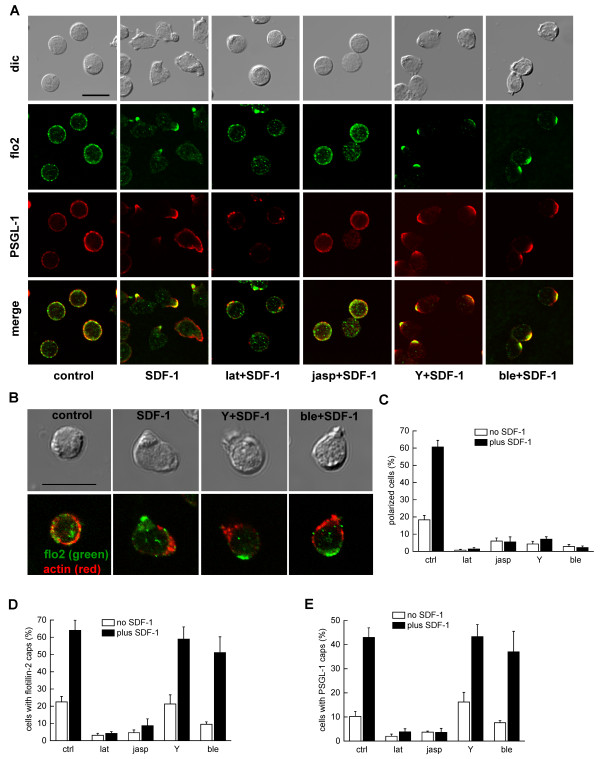
**Role of the cytoskeleton in flotillin- 2 and PSGL-1 capping in human T-lymphoblasts in suspension**. (A,B) After an overnight incubation in the absence of IL-2, T-lymphoblasts were incubated for 45 minutes at 37°C (control) or were preincubated for 30 minutes without or with latrunculin A (lat; 1 μM) or jasplakinolide (jasp; 5 μM) or blebbistatin (ble; 100 μM) or Y-27632 (Y; 10 μM) respectively followed by stimulation with 40 ng/ml of SDF-1 for 15 minutes at 37°C. Cells were then fixed with 10% TCA and (A) double-labeled either for flotillin-2 (flo2; rabbit Ab) and PSGL-1 (murine Ab) or (B) for flotillin-2 (flo2, rabbit Ab) and actin (murine Ab). Bar, 10 μm. (C-E) T-lymphoblasts were treated as described in (A) followed by evaluation of the % of polarized cells with contracted uropods (C); or evaluation of the % of cells with flotillin-2 caps (D) or evaluation of the % of cells with PSGL-1 caps (E). Mean ± s.e.m. of 3-7 independent experiments. At least 40 cells per sample and experiment were analyzed.

T-lymphoblasts were incubated for 16 hours in the absence of IL-2 in order to reduce spontaneous activation in the absence of chemokine. Then cells were stimulated in suspension with the chemokine SDF-1 to induce polarization and migration. Before activation with SDF-1, T-lymphoblasts were mostly spherical with some polarized cells (18 ± 3%, n = 7, average number of total cells assessed per experiment: 70). 15 minutes after addition of the chemokine, 61 ± 4% (n = 7; a total of 58 cells assessed per experiment) of the T-lymphoblasts were fully polarized, featuring a protruding F-actin-rich leading edge and a contracted uropod (Figures 1A-C; 2A-C). We analyzed reorganization of the raft scaffolding proteins flotillin-1 and -2 during cell activation, in comparison with that of the uropod-associated proteins PSGL-1 and activated, phosphorylated ERM proteins.

In the majority of the T-lymphoblasts incubated in the absence of chemokine, flotillin-1 and -2 were located in small randomly distributed plasma membrane-associated patches (Figure 1A-C, left panels). In 22 ± 3% (n = 6; 61 cells assessed per experiment) of cells preformed large caps of flotillin-2 of approximately 2 to 4 μm were detectable. Comparable data were obtained for flotillin-1: in 21 ± 6% (n = 3) of 51 cells assessed per experiment this protein was capped. PSGL-1 showed mainly a punctate random location, with little overlap with flotillin-2 (Figure 1A). Activated ERM proteins phosphorylated on the C-terminal threonine (P-ERM) also showed enrichment in small patches randomly distributed at the plasma membrane rarely overlapping with flotillin-1 (Figure 1B).

Upon stimulation with SDF-1 for 1 minute, the percentage of cells with large caps enriched in colocalizing flotillin-1 and -2 increased markedly (Figure 1C). PSGL-1 colocalized with flotillin caps in 75 ± 4% (n = 3; 68 cells inspected per experiment) of cells with flotillin caps (Figure 1A, middle panels), well comparable to our findings with human neutrophils [16]. P-ERM staining was decreased in part of the cells, but in 51 ± 4% (n = 3; 43 cells inspected per experiment) of the cells with flotillin-2 caps, P-ERM cocapped with flotillin (Figure 1B, middle panels); somewhat in contrast to our findings with human neutrophils where capping of P-ERM lags behind that of flotillins [16]. The flotillin caps always were located in areas opposite to ruffles (Figure 1A-C, middle panels). After 15 minutes of stimulation with SDF-1, flotillin-2 capped in the uropod of 64 ± 6% (n = 5, 58 cells assessed per experiment) of the cells, colocalizing with flotillin-1 (Figure 1C) and also with PSGL-1 and P-ERM (Figure 1A,B, right panels). The fraction of cells with flotillin-2 caps in probes stimulated with SDF-1 for 15 minutes was significantly increased relative to unstimulated controls (3.2 ± 0.3-fold, n = 5, *P *< 0.025) (immunofluorescence pictures: Figure 1A-C, Figure 2A,B; quantitative evaluation: Figure 2D). Flotillin caps were always localized opposite to F-actin-rich ruffles located in the front (Figure 2B). Fifteen minutes after addition of SDF-1, flotillin-1 colocalized with flotillin-2 in 96 ± 5% (n = 3, 57 cells inspected per experiment) of the cells with flotillin uropod caps (values for PSGL-1: 75 ± 11%, n = 4, 58 cells inspected per experiment; P-ERM: 56 ± 6%, n = 4, 50 cells inspected per experiment) (Figure 1A,B). P-ERM staining showed some variation in individual cells, very likely due to variations in the balance of protein kinase and phosphatase activity, as observed also for migrating human neutrophils [19]. Chemokine stimulation of T-lymphocytes thus induces a rapid and marked reorganization and capping of flotillins coinciding with capping of other uropod located proteins. Comparable data were obtained with freshly isolated T-lymphocytes (data not shown).

**Figure 3:**
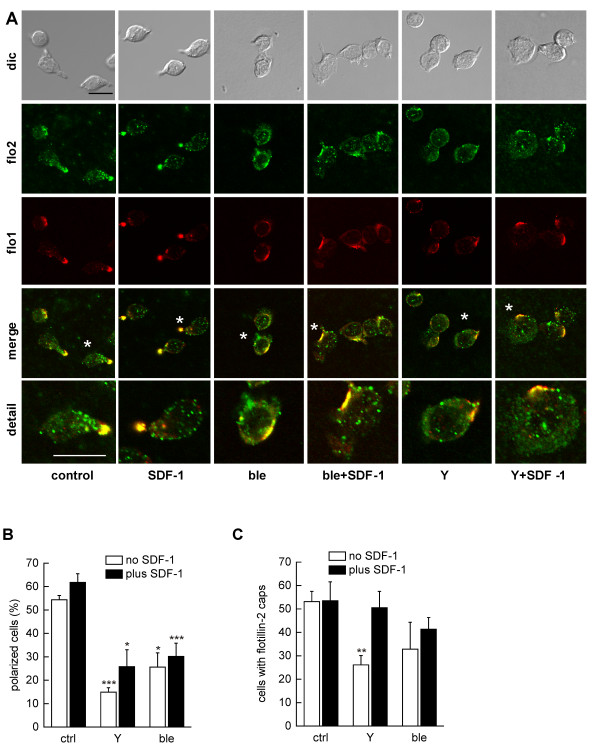
**Roles of myosin II and Rho-kinase in flotillin capping in human T-lymphoblasts adhering to fibronectin**. A: After an overnight incubation in the absence of IL-2, T-lymphoblasts (1.5 × 10^6^cells per well) were placed on fibronectin-coated glass coverslips without or with blebbistatin (ble; 100 μM) or Y-27632 (Y; 10 μM) respectively and allowed to adhere for 30 minutes at 37°C. To part of the samples, 40 ng/ml of SDF-1 was added followed by a further 30 minutes incubation at 37°C (total incubation 60 minutes). Cells were then fixed with 10% TCA and double-labeled for flotillin-2 (flo2; rabbit Ab) and flo1 (murine Ab). Bar, 10 μm. Asterisks indicate cells shown enlarged in the panels labelled "detail". B, C: T-lymphoblasts were treated as described in (A) followed by evaluation of the % of polarized cells with contracted uropods (B); or evaluation of the % of cells with flotillin-2 caps (C). Mean ± s.e.m. of 3-4 independent experiments. At least 40 cells per sample and experiment were analyzed. *P < 0.025, **P < 0.0125, ***P < 0.0005 for differences between data obtained for cells incubated in the absence and presence of inhibitors.

When plated on fibronectin-coated glass in the absence of SDF-1, a much higher percentage of cells showed polarization and flotillin capping in the uropods as compared to cells in suspension, indicating that activation mediated by integrin engagement also induces flotillin uropod recruitment. Incubation with SDF-1 of cells plated on fibronectin induced only a small, statistically not significant increase in the fraction of polarized cells and did not further increase the fraction of cells with flotillin caps (Figure 3A-C).

### Flotillin capping requires the integrity of the actin cytoskeleton but not Rho-kinase and myosin II activity in chemokine-stimulated T lymphoblasts

The actin cytoskeleton has been implicated in contributing to the cellular localization of flotillins [16,18]. Indeed, disruption of the actin cytoskeleton with latrunculin A treatment almost completely suppressed polarization and formation of the SDF-1-induced flotillin-2 and PSGL-1 caps in resting and stimulated T-lymphoblasts in suspension (Figure 2). Cells assumed instead a spherical morphology with a smooth surface. Jasplakinolide, which inhibits depolymerisation of actin filaments and enhances enrichment of F-actin in the cortical cytoskeleton [20], similarly suppressed cell polarization and capping of flotillin-2 and PSGL-1 in resting and stimulated cells (immunofluorescence pictures: Figure 2A,B; quantitative evaluation: Figure 2C-E). These data show that dynamic actin turnover is required for capping of these proteins. Comparable data were obtained for flotillin-1 in T-lymphoblasts and for flotillin-1 and -2 in freshly isolated T-lymphocytes (not shown). We could not study the impact of disruption of actin filaments on cells plated on fibronectin, as cell adhesion was almost completely inhibited by treatment of cells with latrunculin or jasplakinolide (not shown).

Myosin II has been implicated in uropod formation of T-lymphocytes [4]. In agreement with these findings, inhibition of Rho-kinase by Y-27632 or of myosin II activity by blebbistatin almost completely abolished formation of a contracted tail in T-lymphoblasts stimulated by SDF-1 in suspension after inhibitor treatment. The treated cells assumed a spherical morphology with small ruffles on one side. However capping of flotillins and PSGL-1 in SDF-1-stimulated cells was not significantly reduced by pretreatment with Y-27632 or blebbistatin (immunofluorescence pictures: Figure 2A,B; quantitative evaluation: Figure 2C-E). The flotillin- and PSGL-1-rich caps in cells treated with Y-27632 or blebbistatin were always located opposite to F-actin-rich ruffles, on the smooth side of the cells presumably at the site of the former uropod (Figure 2B). Comparable data were obtained for flotillin-1 in T-lymphoblasts, and for flotillin-1 and -2 in freshly isolated T-lymphocytes (not shown).

Similarly, flotillin caps of cells plated on fibronectin were not abolished by pretreatment with blebbistatin or Y-27632 (Figure 3A-C). The effects of these inhibitors on flotillin capping were mostly not statistically significant with the exception of the effect of Y-27632 on cells plated on fibronection in the absence of SDF-1 (inhibition: 48 ± 11%, n = 4, *P *< 0.025). In contrast, pretreatment with Y-27632 or blebbistatin, without or with SDF-1, significantly reduced the fraction of polarized cells (Figure 3B). Cells treated with these inhibitors were more spread (Figure 3A) in agreement with previous findings [21]. Some of the cells exhibited long extended tails, indicating problems with tail retraction (not shown). In conclusion, actin-dependent stimulus-induced coalescence of flotillin- and PSGL-1-containing microdomains in T cells does not require Rho-kinase and myosin II activity.

### Mechanisms of flotillin capping in T lymphoblasts: roles of fatty acid modifications and the putative actin binding site in flotillin-2

**Figure 4:**
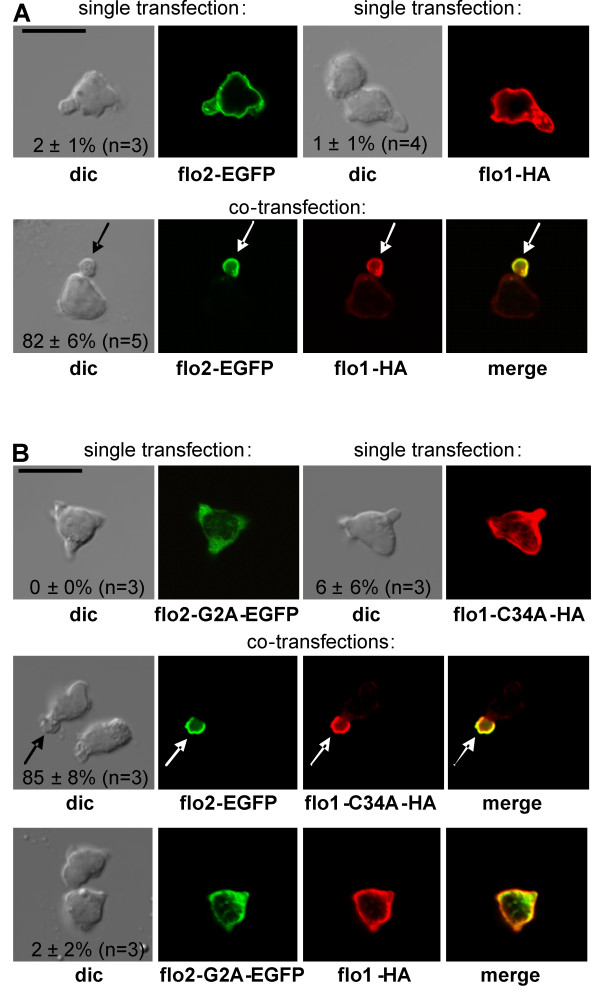
**Roles of flotillin-membrane/raft association in flotillin cap formation in human T-lymphoblasts**. (A) Cells were transfected singly (upper panels) or together (lower panels) with 3 μg of constructs encoding for wild type tagged flotillins (flo1-HA; flo2-EGFP). (B) Cells were transfected singly (upper panels) with constructs encoding for tagged flotillins mutated in residues which carry covalently linked fatty acids (flo1-C34A-HA or flo2-G2A-EGFP), or together with flo2-EGFP and flo1-C34A-HA (middle panel) or together with flo1-HA and flo2-G2A (lower panel). (A,B) 4 hr after transfection cells were stimulated with 40 ng/ml SDF-1, fixed with PFA and the HA-tag was visualized by immunofluorescence staining. Numbers in the panels indicate the fraction of cells with flotillin caps (mean ± s.e.m.) obtained in 3-5 independent experiments. Comparable data were obtained when using mcherry-flotillin-1 instead of HA-tagged flotillin-1 (data not shown). Bar, 10 μm.

We used expression of C-terminally tagged wild type and mutated flotillins to explore mechanisms involved in uropod capping. Wild type flotillins singly expressed in chemokine-stimulated T lymphoblasts showed a uniform membrane association and were also present in intracellular structures located in the uropod of stimulated T lymphoblasts (Figure 4A, upper panels). This is different from location of the endogenous flotillins which are capped in the uropod (Figure 1, 2). Possibly, uropod capping of flotillins depends on heterooligomer formation, and there may not be enough endogenous uncomplexed flotillins available for heterooligomer formation between tagged singly expressed flotillins and endogenous flotillins. According to Solis et al. [22] homooligomerization of singly expressed tagged flotillins is favoured over heterooligomerization with endogenous flotillins. Indeed, in support of a requirement for heterooligomerization of uropod capping, coexpressed tagged flotillin-1 and -2 colocalized in the uropod of 82 ± 6% of the transfected T cells, comparable to the location of the endogenous proteins. Both proteins were almost exclusively restricted to the uropod. (Figure 4A, lower panels).

In order to explore the role of membrane/raft association of flotillins in uropod targeting, we introduced point mutations (G2A in flotillin-2 and C34A in flotillin-1) that have been reported to abolish fatty acid modifications and plasma membrane associations of flotillins [23,24]. Both proteins mutated in this respect, that is, flotillin-2-G2A and flotillin-1-C34A, indeed showed, when transfected singly (Figure 4B, upper panels) or together (not shown), a diffuse cytosolic location, in agreement with previous findings on these mutants in other cell types, indicating a loss of plasma membrane association [23,24]. Interestingly when wild-type flotillin-2 and flotillin-1-C34A were co-transfected, uropod capping of both proteins was as efficient as that of wild-type proteins (Figure 4B, middle panels). In contrast, when flotillin-2-G2A and wild-type flotillin-1 were co-transfected, almost no cap formation occurred (Figure 4B, lower panels). In summary the data shown in Figure 4 strongly suggest that heterooligomerization of flotillin-1 and -2 is required for uropod capping and that membrane association of flotillin-2, but not of flotillin-1, is necessary for targeting of the heterooligomers to the uropod.

**Figure 5:**
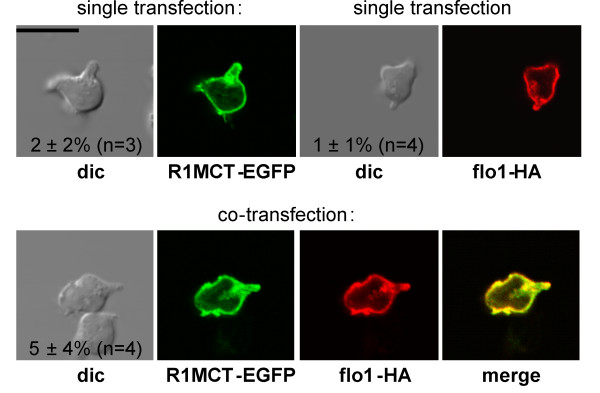
**Role of flotillin-F-actin interaction in flotillin cap formation in human T-lymphoblasts**. Cells were transfected singly (upper panels) or together (lower panels) with a construct encoding for flotillin-2 lacking the F-actin binding domain (R1MCT-EGFP) and flo1-HA, as indicated. 4 hr after transfection cells were stimulated with 40 ng/ml SDF-1, fixed with PFA and the HA-tag was visualized by immunofluorescence staining using a murine monoclonal anti-HA antibody. Numbers in the panels indicate the fraction of cells with flotillin caps (mean ± s.e.m.) obtained in 3-4 independent experiments.

We further analyzed the role of the F-actin binding site located in the N-terminal half of flotillin-2. A deletion construct lacking amino acids 31-183 of flotillin-2 (R1MCT) still retains the ability to interact with membranes and with flotillin-1 but does not colocalize anymore with F-actin when expressed in cells [18]. This singly expressed deletion construct showed a uniform membrane association and was also present in vesicles (Figure 5, upper left panels). When coexpressed with wild type flotillin-1, this location was not changed, little capping was observed and both proteins colocalized, suggesting that direct F-actin interaction is indeed required for flotillin uropod capping in T-lymphocytes (Figure 5, lower panels).

### Stability of flotillin caps in T-lymphoblasts

**Figure 6:**
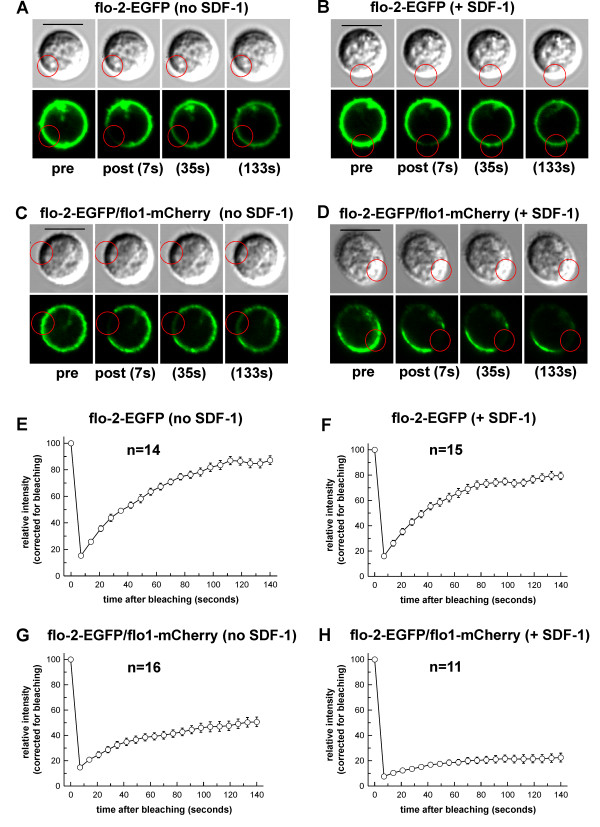
**FRAP analysis of the lateral mobility of flotillin-2-EGFP, expressed without or with flotillin-1-mCherry in T-lymphocytes**. Freshly isolated human T-lymphocytes were transfected with either flotillin-2-EGFP alone (A,B,E,F) or with flotillin-2-EGFP and flotillin-1-mCherry together (C,D,G,H), followed by a 4 hour incubation prior to FRAP analysis. Just before the FRAP analysis, cells were incubated for 15 minutes in the absence (left panels) or presence (right panels) of 40 ng/ml SDF-1, followed by bleaching of EGFP using excitation at 473 nm for all samples. Images of representative cells 2 seconds before (pre) and 7- 133 seconds after (post) completion of bleaching are shown, as indicated. Bleached regions of interest (ROI) are shown as red circles. Bar, 10 μm. (E-H) Fluorescence recovery time course after photobleaching of EGFP (mean ± s.e.m.) averaged from 11-16 cells derived from 3-4 independent experiments, as indicated in the panels. The prebleaching intensity was set to 100 in panels E-H. The values were corrected for bleaching due to acquisition of frames assessed in control ROIs corresponding to non-bleached regions analyzed in the same cells where localized bleaching was performed (not shown in A-D). Note that these FRAP experiments were carried out at 20°C, where cells are not any more polarized but still feature flotillin caps. At 37°C rapid shape changes and migration precluded accurate bleaching, acquisition and evaluation of FRAP data.

We wanted to compare lateral mobility of singly transfected membrane-associated flotillin-2-EGFP with that of flotillin-2-EGFP co-assembled with flotillin-1-mCherry in large caps. We therefore carried out FRAP (fluorescence recovery after photobleaching) in transfected T-lymphoblasts stimulated with SDF-1. Note that these FRAP experiments were carried out at 20°C as rapid shape changes and migration occurring at 37°C precluded accurate bleaching, acquisition and evaluation of FRAP data. At 20°C cells were not any more polarized but the flotillin caps persisted for a longer time. FRAP experiments demonstrated a relatively high mobility of singly transfected flotillin-2-EGFP at the plasma membrane. The flotillin-2-EGFP fluorescence recovered with a half time of approximately 30 seconds and the mobile fraction M_F _corresponded to 0.80 ± 0.06 (a total of 7 cells analyzed from 3 independent experiments) (Table 1). Comparable data were obtained for cells transfected with flotillin-1-EGFP (Table 1). In contrast, in caps consisting of flotillin-1-mCherry and flotillin-2-EGFP, almost no recovery of fluorescence within the time of observation was observed. A mobile fraction of only M_F _= 0.06 ± 0.02 (7 cells; 3 independent experiments) (Table 1) suggests nearly complete immobilization of flotillin-2 in the caps. Comparable data were obtained for flotillin-1-EGFP cotransfected with flotillin-2-mCherry (Table 1). We now investigated the impact of chemokine stimulation on the lateral mobility of flotillins. To this end we used transfected freshly isolated T-lymphocytes, as the basal level of flotillin cap formation in these cells in the absence of chemokine is lower as compared to T-lymphoblasts. As shown in Figure 6 and Table 1, flotillin-2-EGFP transfected alone is very mobile irrespective of the presence of chemokine. However, in cells expressing both flotillin isoforms, mobility of flotillin-2-EGFP was markedly decreased by 53 ± 7% (n = 4, *P *< 0.01) already in the absence of chemokine as compared to singly transfected cells (Table 1). Chemokine addition resulted in almost complete immobilization of flotillin-2-EGFP when co-expressed with flotillin-1 (69 ± 7% reduction of M_F _(n = 4, *P *< 0.005), as compared to cells coexpressing both flotillins and incubated in the absence of chemokine) (Table 1). Our results strongly suggest that the lateral mobility of flotillins is regulated a) by heterooligomer formation and b) by chemokine stimulation.

**Table 1:** FRAP analysis of the lateral mobility of flotillins in T-lymphocytes

Transfection	Cell type	M_F _- SDF-1 (n)	M_F _+ SDF-1 (n)
flo1-EGFP	T-lymphoblast	n.d.	0.88 ± 0.05 (12)
flo1-EGFP/flo2-mCherry	T-lymphoblast	n.d.	0.04 ± 0.01 (10)
flo2-EGFP	T-lymphoblast	n.d.	0.80 ± 0.06 (7)
flo2-EGFP/flo1-mCherry	T-lymphoblast	n.d.	0.06 ± 0.02 (7)
flo2-EGFP	Freshly isolated T-cells	0.79 ± 0.04 (14)	0.71 ± 0.03 (15)
flo2-EGFP/flo1-mCherry	Freshly isolated T-cells	0.38 ± 0.04 (16)	0.11 ± 0.02 (11)

FRAP analysis involving local bleaching of EGFP was carried out as described in the legend of Figure 6 with cells preincubated in the absence or presence of SDF-1, as indicated. The mobile fraction (M_F_) was calculated for each individual cell and then averaged from a total of n = 7-16 cells, as indicated (mean ± s.e.m.) (data obtained in 2-4 independent experiments). N.d. = not done.

In a separate series of experiments we studied the role of F-actin in immobilizing flotillins in freshly isolated T-lymphocytes. As shown in Table 2, pretreatment of cells with latrunculin A markedly and significantly increased the lateral mobility of co-expressed flotillins in cells exposed to SDF-1 6.3 ± 1.4-fold (n = 3, *P *< 0.001), whereas the impact of this treatment on flotillin mobility in cells not stimulated by chemokine was smaller (2.1 ± 0.5-fold; n = 3, *P *< 0.05). In agreement with the results obtained with latrunculin on cells transfected with wild type flotillins, the flotillin-2 mutant R1MCT lacking the F-actin binding site showed a mobility comparable to that of singly transfected wild type flotillins irrespective of the presence of co-expressed wild type flotillin-1 and SDF-1 (Table 3).

**Table 2:** Impact of pretreatment with latrunculin A on the lateral mobility of flotillin-2 cotransfected with flotillin-1 in freshly isolated T-lymphocytes

Transfection	Pretreatment	M_F _- SDF-1 (n)	M_F _+ SDF-1 (n)
flo2-EGFP/flo-1-mCherry	No pretreatment	0.25 ± 0.03 (11)	0.11 ± 0.04 (12)
flo2-EGFP/flo1-mCherry	Preincubation for 30 min with latrunculin A (1 μM)	0.46 ± 0.07 (12)	0.66 ± 0.07 (11)

FRAP analysis involving local bleaching of EGFP was carried out as described in the legend of Figure 6 with cells preincubated in the absence or presence of SDF-1, and without or with pretreatment with latrunculin A as indicated. The mobile fraction (M_F_) was calculated for each individual cell and then averaged from a total of n = 11 - 12 cells observed in 3 independent experiments, as indicated (mean ± s.e.m.).

**Table 3:** Lateral mobility of the flotillin-2 mutant lacking the actin binding domain (R1MCT) expressed in freshly isolated T-lymphocytes without or with wild type flotillin-1-mCherry

Transfection	M_F _- SDF-1 (n)	M_F _+ SDF-1 (n)
R1MCT	0.72 ± 0.05 (15)	0.79 ± 0.06 (14)
R1MCT/flo1-mCherry	0.65 ± 0.06 (14)	0.79 ± 0.05 (15)

FRAP analysis involving local bleaching of EGFP was carried out as described in the legend of Figure 6 with cells preincubated in the absence or presence of SDF-1 as indicated. The mobile fraction (M_F_) was calculated for each individual cell and then averaged from a total of n = 14 - 15 cells observed in 3 independent experiments, as indicated (mean ± s.e.m.).

These findings suggest that SDF-1 markedly enhances anchoring of flotillin-rich membrane microdomains to the underlying F-actin network which results in almost complete immobilization of these proteins.

We further tested a possible role of myosin II in regulating flotillin mobility using FRAP of blebbistatin-treated cells. In this case bleaching of EGFP could not be used, as illumination of cells incubated with blebbistatin at 450-490 nm (but not at 510-560 nm) has been shown to cause dose-dependent inhibition of cell motility and cell death due to generation of toxic blebbistatin products [25]. We therefore used bleaching of mCherry-tagged flotillins at 559 nm for these experiments. As shown in Table 4, blebbistatin treatment did not significantly affect flotillin-2 mobility, irrespective of the presence of SDF-1. Comparable data were obtained for flotillin-1 tagged with mCherry (data not shown). Myosin II thus appears not to be involved in immobilization of flotillin-rich membrane microdomains.

**Table 4:** Impact of pretreatment with blebbistatin on the lateral mobility of flotillin-2 cotransfected with flotillin-1 in freshly isolated T-lymphocytes

Transfection	Pretreatment	M_F _- SDF-1 (n)	M_F _+ SDF-1 (n)
flo2-mCherry/flo-1-EGFP	No pretreatment	0.43 ± 0.05 (11)	0.11 ± 0.03 (13)
flo2-mCherry/flo1-EGFP	Preincubation for 30 min with blebbistatin (100 μM)	0.49 ± 0.06 (9)	0.09 ± 0.02 (10)

FRAP analysis involving local bleaching of mCherry was carried out as described in the legend of Figure 6 with cells preincubated in the absence or presence of SDF-1, and without or with pretreatment with blebbistatin as indicated. The mobile fraction (M_F_) was calculated for each individual cell and then averaged from a total of n = 9 - 13 cells observed in 3 independent experiments, as indicated (mean ± s.e.m.).

### Transfection of T-lymphoblasts with flotillin-G2A-HA impairs cell polarization and capping of endogenous flotillin-1 and PSGL-1

**Figure 7:**
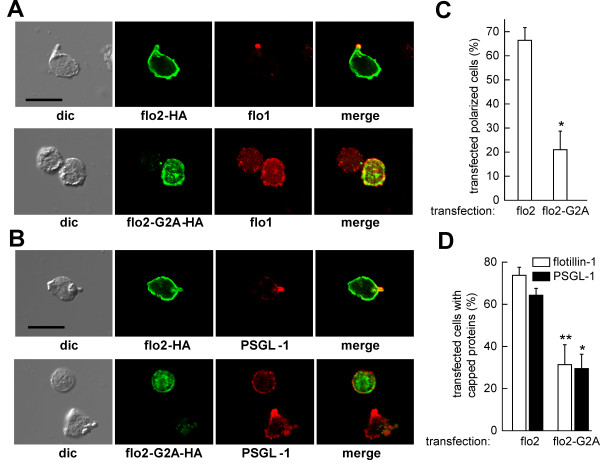
**Impact of transfection of T-lymphoblasts with flotillin-2-G2A on cell polarization and capping of uropod components**. (A,B) T-lymphoblasts were incubated for 22 hours in the absence of IL-2, followed by transfection with 2 μg of constructs encoding for HA-tagged wild-type flotillin-2 (flo2) or flotillin-2-G2A (flo2-G2A). Five hours after transfection, IL-2 was added followed by a further 17 hour incubation, stimulation with SDF-1 (15 minutes, 40 ng/ml), TCA-fixation and double-labeling for HA-tagged wild-type or mutated flotillin-2 (flo2-HA; flo2-G2A-HA) and endogenous flotillin-1 (flo1) (A) or HA-tagged wild-type or mutated flotillin-2 and endogenous PSGL-1 (B), using a polyclonal rabbit anti-HA antibody and monoclonal murine antibodies directed against flotillin-1 and PSGL-1. Bar, 10 μm. (C,D) T-lymphoblasts were treated as described for (A,B), and the amount of polarized transfected cells (n = 3) (C) or the amount of transfected cells with capped endogenous flotillin-1 (n = 4) or PSGL-1 (n = 3) (D) were evaluated (mean ± s.e.m.). 30-50 transfected cells were analyzed per sample and experiment. *P < 0.025, **P < 0.0125 for differences between data obtained for cells transfected with HA-tagged wild type or mutated flotillin-2.

In order to unravel the role of flotillins in T-lymphocyte migration and polarization we attempted to down-regulate flotillin expression using nucleofection with siRNA in T-lymphoblasts. However we only achieved maximally 50% downregulation of flotillin-2 and -1. Uropod formation was not significantly impaired (data not shown) suggesting that the residual flotillin proteins are sufficient to maintain cell polarization. We therefore tested the impact of transiently overexpressing flotillin-2-G2A in T-lymphoblasts, as compared to wild type flotillin-2, on uropod formation and capping of endogenous flotillin-1 and the uropod-associated protein PSGL-1. Flotillin-2-G2A has been suggested to act as a dominant-negative mutant in HeLa cells [26]. It may act by forming unproductive complexes with endogenous flotillin-1, thus preventing flotillin-1 in forming functional heterooligomers with endogenous flotillin-2. This could lead to displacement of endogenous flotillins from the uropod and impaired cell polarization. In order to reduce as much as possible endogenous stable flotillin caps, cells were preincubated in buffer lacking IL-2 prior to transfections. We first wanted to know whether overexpression of flotillin-2-G2A indeed displaces endogenous flotillin-1 from the uropod. Whereas the majority of cells overexpressing wild-type flotillin-2 showed clear-cut uropod-located flotillin-1 caps, we observed a statistically significant 57 ± 13% (n = 4; *P *< 0.0125) reduction of flotillin-1 caps in cells expressing mutant flotillin-2 (immunofluorescence pictures: Figure 7A; quantitative evaluation: Figure 7D). Similarly, capping of PSGL-1 was significantly reduced in cells expressing the flotillin-2 G2A mutant by 53 ± 11% (n = 3; *P *< 0.025) (immunofluorescence picture: Figure 7B; quantitative evaluation: Figure 7D). Importantly, cell polarization, that is, cell elongation and front-tail polarity with a clearly defined uropod was also markedly reduced in cells expressing mutant flotillin-2, by 66 ± 15% (n = 3; *P *< 0.025) (Figure 7A-C), supporting indeed a role of flotillins in T-lymphocyte polarization, similar to recently published work on neutrophils [17].

## Discussion

Flotillin-1 and -2 have previously shown to be enriched in membrane-associated caps in Jurkat T-cells and Molt4 cells and in the uropods of polarized human T-lymphoblasts, monocytes, CD34-positive cells and human neutrophils [12-14,16]. This polarized location of flotillins appears to be restricted to hematopoietic cells as it does not occur in for example polarized epithelial cells [14]. In murine neutrophils, flotillins have recently been shown to be required for uropod formation and migration through matrigel [17]. We have now investigated mechanisms and functional role of flotillin capping in human T-lymphoblasts.

### Stimulus-dependent flotillin capping in T-lymhocytes

We show that preformed large flotillin caps of 2-4 μm are present in 22 ± 3% (n = 6) of human primary T lymphoblasts incubated in suspension prior to activation with chemokine, and that the number of such caps is markedly and rapidly increased within one minute upon chemotactic stimulation of cells in suspension. We further show that flotillin-1 and -2 are localized almost exclusively in caps at the tip of the uropod in the rear of polarized cells, partially colocalizing with other uropod resident molecules such as P-ERM and PSGL-1. Stimulus-dependent coalescence of flotillin-containing microdomains is thus an early event during T cell activation, and such domains may mark the site of the future uropod, comparable to our previous findings with human neutrophils [16]. Similar data were obtained for cells plated on fibronectin, although there formation of flotillin caps was enhanced already in the absence of SDF-1, presumably by integrin-mediated cell activation.

### Role of the cytoskeleton in flotillin cap formation

The actin cytoskeleton is required for organization and function of membrane microdomains and redistribution of raft-associated proteins in the uropod of leukocytes [27-29]. Suppression of myosin II activation by the Rho-kinase inhibitor Y-27632 or the myosin II inhibitor blebbistatin, or myosin IIA knockout inhibits chemokine-induced uropod formation of T-lymphocytes treated in suspension, promotes long non-retracting tails in T-cells plated on substrate, enhances cell spreading and markedly attenuates T cell migration and chemotaxis in vitro and in vivo [21,30-34]. Moreover retrograde cortical flow of actin filaments depending on myosin II contractility been implicated in accumulation of actin-attached surface receptors in the uropod of T-cells [1]. We thus explored the contribution of actin and myosin II to the capping of flotillin isoforms in the uropods of T-lymphoblasts. We show that this event depends on a functional actin network, but is not abolished by inhibition of Rho-kinase or myosin II activity, suggesting that retrograde cortical flow of F-actin mediated by myosin II very likely is not involved. Rather, flotillins appear to control myosin II activity [17]. Our findings in T-lymphocytes are comparable to our data obtained in human neutrophils [16].

Actin could organize flotillin caps indirectly via other proteins or by direct interactions with flotillins. Indeed, the SPFH domain (as 41-183) of flotillin-2 has been shown to interact directly with F-actin in vitro, and flotillin-2 lacking this domain (the deletion mutant R1MCT) does not anymore colocalize with F-actin when expressed in HeLa cell and N2a cells [18]. The mutant R1MCT should still retain the capacity to form heterooligomers as it contains the coiled-coil stretch required for heterooligomerization [22]. We now show that co-expression of R1MCT with wild type flotillin-1 does not result in cap formation, and that R1MCT is highly mobile in the plane of the membrane irrespective of cell stimulation and co-expression of wild type flotillin-1, suggesting that direct F-actin interaction with flotillin-2 is required for targeting the heterooligomers to the uropod and stabilization in the uropod (Figure 5). As rather a large part of the protein (amino acid residues 30-184; 36%) was deleted, it cannot be excluded that the abolishment of uropod recruitment is due to a loss of interactions with proteins other than F-actin. It is unclear why a direct interaction with F-actin results in flotillin raft coalescence selectively in the relatively F-actin-poor uropod and not also in F-actin-rich ruffles at the leading edge. A possibility could be a specific interaction with a stable F-actin population which has been shown to be enriched in the uropod of polarized neutrophil-like HL-60 cells and zebrafish neutrophils, as opposed to a dynamic F-actin pool present in the front [35,36]. Future studies should show, if such a stable F-actin population is also present in the uropod of polarized T-lymphocytes and if it colocalizes with flotillins.

### Heterooligomer formation and capping of flotillins

According to previous data, EGFP-tagged flotillin-2 singly expressed in Jurkat T-cells forms stable large caps [12,13]. However this is not the case in naïve T-cells and human T-lymphoblasts where we observed uniform membrane association of singly expressed EGFP-flotillin-2. The same was observed for singly expressed HA-tagged flotillin-1 (Figure 4A, 7A). In contrast co-expressed flotillin-1 and -2 formed stable membrane-associated caps in almost all chemokine-stimulated, transfected cells (Figures 4A, 6D). These data suggest, that heterooligomer formation is required for uropod targeting of flotillins in T cells and that singly expressed flotillins do not heterodimerize extensively with endogenous flotillins. Indeed Solis et al. [22] observed that, when expressed in neuro2a cells, homooligomerization of singly expressed tagged flotillins is favoured over heterooligomerization with endogenous flotillins. They explain this with faster de novo synthesis of the tagged proteins. An explanation for the differences in data obtained in Jurkat cells and T-cells could be that there is more free endogenous flotillin-1 available for heterooligomer formation with exogenous flotillin-2 in Jurkat cells as compared to T cells. Alternatively, complex formation of flotillin-1 and -2 is required for capping only in human peripheral blood T-cells but not in Jurkat cells. Our data agree with the recent work of Ludwig et al. [17] where it could be shown that flotillin-rich microdomains are absent in mice lacking flotillin-1.

### Role of covalent fatty acid modifications of flotillin-1 and -2 in formation of uropod-associated flotillin caps

Fatty acid modifications on cysteine 34 in flotillin-1 and glycine 2 in flotillin-2 have been shown to be required for membrane association of flotillin-1 and -2 in other cell types [23,24]. We now show that fatty acid modification of flotillin-2, but not of flotillin-1 is required for uropod targeting in T-lymphocytes using point mutations of these amino acids to alanine. Wild-type membrane-associated flotillin-2 can thus recruit cytosolic flotillin-1-C34A to the uropod, whereas wild-type flotillin-1 cannot recruit flotillin-2-G2A. These novel findings suggest that membrane association of flotillin-2 is sufficient and necessary for raft/membrane localization of the heterooligomers.

In summary, our data strongly suggest that heterooligomer formation and plasma membrane association of flotillin-2 are prerequisites for uropod recruitment of flotillins in T lymphocytes. Possibly heterooligomer formation induces a conformational change in the proteins facilitating further interactions with other binding partners.

### Lateral mobility of membrane-associated flotillins

We analyzed the lateral mobility of tagged flotillins expressed in T-lymphocytes using FRAP. Our data show that singly transfected flotillins are highly mobile and show a uniform plasma membrane association irrespective of the presence or absence of chemokine. When coexpressed, the mobility of flotillins is markedly reduced even in the absence of chemokine. In the presence of chemokine, tagged coexpressed flotillins coalesce in large uropod-located caps. Flotillins in these large caps are almost completely immobilized (Figure 6, Table 1). Mobility of flotillins in especially chemokine-treated cells was markedly enhanced by latrunculin-mediated disruption of F-actin, almost to levels of singly transfected molecules. The impact of F-actin disruption on flotillin mobility was much smaller (but still significant) in cells not exposed to chemokine. Myosin II inhibition did not affect flotillin mobility in resting and stimulated cells. We propose based on these data, that flotillin heterooligomers in the absence of chemokine form small randomly distributed microdomains. These microdomains are partially immobilized by interaction with either a subpopulation of F-actin relatively insensitive to latrunculin or with unknown cellular components. Our data support the notion that complete immobilization of flotillins in the large, stimulus-dependent caps occurs due to stimulus-dependent enhancement of actin polymerization and interaction of the flotillin rafts with a pool of stable F-actin (see above), independent of myosin II activity.

### Functional roles of flotillin caps in T-lymphocytes

We propose that flotillins act as scaffolding proteins structuring the uropod and regulating signaling molecules. Indeed our data using overexpression of the flotillin-2-G2A mutant suggest an important role in uropod formation and recruitment of PSGL-1 (Figure 7), correlating well with data recently published on murine neutrophils [17]. Early during lymphocyte activation, prior to uropod formation, flotillins may mark that part of the plasma membrane that will later on, based on myosin II contractility, develop into a uropod. These flotillin caps, stably linked to F-actin, may prevent protrusion formation in that area and recruit other uropod-located proteins, possibly enhancing local rear signaling and myosin II activation [17,37]. Moreover flotillins have been implicated in clathrin-independent endocytosis of GPI-anchored proteins [38,39]. T cells have been shown to contain large clathrin-based endocytic platforms at the uropod which have been implicated to contribute to chemotaxis [34] Similarly flotillins could be involved in clathrin-independent endocytosis of specific molecules involved in polarization and chemotaxis.

## Conclusions

In conclusion, we provide novel evidence demonstrating that formation of large stable uropod-associated flotillin caps in T-lymphocyte is markedly enhanced by cell stimulation. We show, that these events depend on actin dynamics but not on actomyosin contractility. Uropod capping of flotillins moreover requires coexpression of flotillin-1 and -2, and thus very likely flotillin heterooligomer formation, as well as the presence of a domain in flotillin-2 implicated in direct F-actin interactions and plasma membrane association of flotillin-2, but not -1. We show that the lateral mobility of flotillins in the plasma membrane is reduced by co-expression of both flotillins and is further almost abolished by cell stimulation, depending on a functional actin network. Overexpression of mutant flotillin-2GA unable to interact with rafts reduces capping of endogenous flotillin-1 and PSGL-1 and impairs chemokine induced cell polarization. Our findings suggest an important functional role of flotillins in uropod structuring. Open questions concern possible direct interaction partners of flotillins in the uropod of polarized lymphocytes, and the exact molecular mechanisms involved in stimulus-dependent flotillin activation, capping and uropod targeting.

## Competing interests

The authors declare that they have no competing interests.

## Authors' contributions

SA and JH participated in the experiments with the inhibitors; SA prepared the flotillin constructs and carried out the transfections of tagged flotillins; SM carried out and analyzed the FRAP experiments, JR carried out initial experiments on purification and immunofluorescence staining of T-lymphocytes, VN conceived and designed the experiments, analyzed the data and wrote the manuscript. All authors read and approved the final manuscript.
